# Practices and discourses of ubuntu: Implications for an African model of disability?

**DOI:** 10.4102/ajod.v6.292

**Published:** 2017-01-31

**Authors:** Maria Berghs

**Affiliations:** 1 Private, Belgium

## Abstract

**Background:**

Southern African scholars and activists working in disability studies have argued that *ubuntu* or *unhu* is a part of their world view.

**Objectives:**

Thinking seriously about *ubuntu,* as a shared collective humanness or social ethics, means to examine how Africans have framed a struggle for this shared humanity in terms of decolonisation and activism.

**Method:**

Three examples of applications of *ubuntu* are given, with two mainly linked to making explicit *umaka.* Firstly, *ubuntu* is linked to making visible the invisible inequalities for a common humanity in South Africa. Secondly, it becomes correlated to the expression of environmental justice in West and East African countries.

**Results:**

An African model of disability that encapsulates *ubuntu* is correlated to how Africans have illustrated a social ethics of a common humanity in their grassroots struggles against oppression and disablement in the 20th century. *Ubuntu* also locates disability politically within the wider environment and practices of sustainability which are now important to the post-2105 agenda, Convention on the Rights of Persons with Disabilities (CRPD) and the (UN) Sustainable Development Goals linked to climate change.

**Conclusion:**

A different kind of political action linked to social justice seems to be evolving in line with *ubuntu*. This has implications for the future of disability studies.

## Introduction

The literature about disability and decolonisation in disability studies has primarily been shaped by academics located in a minority world or the Global North (i.e. Connell 2011; Grech 2015; Meekosha 2011), even though this is slowly changing (Mji et al. 2011; Opini 2016). Despite this state of affairs, decolonisation does not have to be epistemologically nor ontologically located within the imaginaries of elite northern academics. Examining the social and cultural history of the African continent tells a completely different story and illustrates how Africans have formulated and shaped theories as well as actions of decolonisation *and* relationships to disability in their own epistemological and transnational terms. As such, this paper does not seek to decentre but argues that theory and the links to activist practice have always been located in the global south even in its hybrid or transnational forms (Comaroff & Comaroff 2012; de Sousa Santos 2015:4). It seeks to examine the implications of the continent’s rich and diverse legacy, in terms of activism and social movements, to celebrate and learn from.

In Africa, there is a multiplicity of interpretations of ‘disability’ from: depictions found in oral histories, music, dance, ritual, (secret) society practices of different ethnic groups; the colonial and postcolonial histories of medical segregation and prevention; how differing religions, evangelical and missionary services and their organisations understand disability; the measurements and international standards set by international organisations such as the World Health Organization, as well as how these institutions demarcate differences to disease, illness and impairment; the influence of the disability movements that ascribe to various definitions of disability and their advocacy on international and national policy agendas linked to human rights, development and now sustainability; the ‘persons with disabilities’ definition advocated by the United Nations (UN) and enshrined in legislation in the Convention on the Rights of Persons with Disabilities (CRPD) and how that becomes translated nationally and implicated bureaucratically in the machinery of the state; the theories and models of disability that international organisations, civil societies and non-governmental organisations are working with; how radio, TV and social media are impacting disability; and how everyday popular culture, music and the arts define and people understand what disability entails.

In this paper, I will examine disability in terms of *ubuntu*. Southern African scholars and disability activists have argued that *ubuntu* or *unhu* is a part of their world view – a philosophy of shared collective humanness and responsibility (i.e. Chataika et al. 2015; Mji et al. 2011; Opini 2016). I will elucidate what *ubuntu* means and then examine how it connects to social action. The aim is to shine a light on what African discourses and practices can teach about disability. How disability becomes defined and framed, implications of this for people’s lived experience and what we can learn about the future direction of disability studies.

## *Ubuntu*: Implications for understanding disability

*Ubuntu* is an African humanist and ethical world view where disability, as part of a common humanity, is necessarily part of what makes us human. In the Zulu language, the expression is *umuntu ngumuntu ngabantu* which translates as, ‘a person is a person through other persons’ (Shutte 1993:46). Louw (1998), quoting Van der Merwe (1996:1), argues that another reading of this phrase is, ‘A human being is a human being through (*the otherness of*) other human beings’. At the heart of *ubuntu* is a respect for a diversity of what it means to be human (Eze 2008). Van der Merwe (1996) argues that *ubuntu* is not just descriptive but also a normative ethical claim about how we *should* behave towards others and how to become human.

In an *ubuntu* model of disability, impairment becomes cognitive, sensory, mental, physical (inclusive of biological) and spiritual diversity that can have a multitude of shared meanings that society, as human collective, constantly (re) make together. *Ubuntu* can change over time and recognises difference of experiences of diversity of humanness (as positive or negative), which are part of our shared humanity. This includes interpersonal relationships and reactions, such as affect (Livingston 2008). Disablement happens when that otherness or diversity becomes a difference predicated as inhuman, for example, in that a person is viewed as threatening the social order, kinship relations or is viewed as morally outside the realm of what it socially means to be human. In order to understand humanness, Murove (2004) and Le Grange (2012) correlate *ubuntu* to *ukama,* a feeling of relatedness or interdependence, but argue that this includes the wider environment, the biophysical world. Thus, to understand humanness and relatedness means to understand that what it means to be human is part of a biologically (Le Grange 2012) and spiritually diverse ecology (Murove 2004). According to Murove (2004), *ubuntu* encompasses a spiritual interdependence and respect, for example, towards the ancestors and sacred found in our ecosystems, such as burial grounds or totemic animals. This opens up disablement to also include a diversity that is wider than just biological impairment orientated but is also linked to understanding how the spiritual and ecological are linked together. Disablement is also not fixed but can be rectified through taking on the ethical responsibilities of shared collective actions. Oppression occurs when an individual, collective or the (physical or spiritual) environment of what allows *ubuntu*, our common humanity, is being threatened by inhuman actions that cause harm, such as forms of exploitation, degradation or violence.

Thinking seriously about *ubuntu,* as a shared collective humanness or social ethics, means to examine how people framed a struggle for this shared humanity in terms of a past and present history of decolonisation and activism (Louw 1998). *Ubuntu* has mostly been analysed in terms of how it leads to respectful community dialogue and consensus for a restorative justice, for example, in the South African post-apartheid context (Louw 2006). An application of *ubuntu* in terms of restorative justice, for instance, linked to the South African Truth and Reconciliation Commission, was to guarantee that a victim gains an apology for harm done and the perpetrator asks for forgiveness and is reintegrated back into a community. The victim and perpetrator are viewed as interlinked, part of *ubuntu,* with both necessitating healing. Ultimately, they are implicated in terms of the strength of the group that depends on a social ethics of humanness or *ubuntu* that has been harmed, because of a history of colonialism and apartheid violence.

Yet, critics have also argued that *ubuntu* places the needs of group solidarity first and does away with individual criticism of the community or even real justice for both victims and perpetrators (Louw 2006). This could be because an understanding of how *ubuntu* relates to *umaka* is missing in that restorative justice does not actually restore the environment, which is part of humanness. Viewing *ubuntu* as a social ethics upholding humanity outside and inside the South African context illustrates what restorative actions look like to prevent disablement. It is *ubuntu* that allows the identification of African social ethical discourses and practices countering colonialism, disablement and oppression. A social ethics of *ubuntu* has its roots in collective social action for a shared humanity against oppression and injustice. In the African context, this has taken on multiple forms. In this paper, I will give three examples of applications of *ubuntu,* with two mainly linked to making explicit *umaka.* Firstly, I examine how *ubuntu* became linked to making visible the invisible inequalities for a common humanity in South Africa and then turn to expression of environmental justice in West and East African countries. Lastly, the paper turns to establish a dialogue on what the history and understanding of practices of *ubuntu* can tell us about future directions in disability studies.

## Making Visible the Invisible: A Common Humanity in South Africa?

Above all, we want equal political rights, because without them our disabilities will be permanent. I know this sounds revolutionary to the whites in this country, because the majority of voters will be Africans. This makes the white man fear democracy. From Mandela, Nelson (1978) The Struggle Is My Life. (Finkelstein 2001a, p.2.)

Nelson Mandela is often viewed as the personification of what *ubuntu* should mean, especially in terms of his actions towards reconciliation in post-apartheid South Africa. In the above quote, taken from a speech he gave before he was imprisoned, he notes how the system of apartheid and inequality leads to inhumanity. An inhumanity that is predicated on difference of skin colour as impairment. An impairment that Mandela knew was an illusion. The disability that he refers to is the inability to live autonomously and have rights of citizenship. This speech struck a chord with many African activists fighting for their independence and freedom. It also galvanised people who faced discrimination based on differences they felt were artificial such as age, religion, ethnicity, sexuality, gender and so on. Much consciousness rising from the 1960s onwards focused on making visible these invisible injustices that people experienced and this was revolutionary. The South African history of activism in this respect has been fundamental in revising the definition and meaning we give to ‘disability’, successful organisation around rights and embodying a language of a common humanity in *ubuntu.* I examine three ways in which this has been done in South Africa’s history; (1) in influencing the social model of disability, (2) disability rights activism and the unification of social movements, and (3) a shared humanity.

### Influence on the social model of disability

Mandela’s speech was also important to the disabled activist and academic, Vic Finkelstein, who in the 1960s was involved in South Africa’s anti-apartheid movement and struggle for independence (see Berghs 2015). For him, disability also meant a loss of freedom and inaccessible rights of citizenship. Finkelstein was one of the founders of United Kingdom’s Union of the Physically Impaired Against Segregation (UPIAS) in 1972 with Paul Hunt. UPIAS was an organisation run by disabled activists that also lay at inception of the social model of disability. The social model makes a difference between impairment (physical, cognitive, sensory) and the disability as experience of oppression caused by society. Finkelstein recounts that it was his Jewish background, witnessing of apartheid and experiences of imprisonment as disabled activist that lead to this understanding of disability (Finkelstein 2001a, 2001b). Until that point, he had accepted an understanding of ‘disability’ in terms of medical and charitable approaches. Yet, while he was imprisoned he noted that the apartheid state was forced to make the prison accessible to him and thus the state could remove barriers to ensure his inclusion and independence. When threatened with violence that would break his legs, he noted he was already in a wheelchair. When freed from prison, he was given a banning order but noted that everything (i.e. health, housing, transport, social activities) was already socially inaccessible to him so the banning order was expedient (Finkelstein 2001a, 2001b).

In keeping with *ubuntu,* Mandela questions the very notion of impairment but Finkelstein does not. From personal experience, he knew that impairment could be tragic but he felt this did not explain his segregation from society; a society that seemed to have been created for able-bodied people with different abilities (Finkelstein 2001b). He interpreted disability as social oppression that was imposed on top of his personal (tragic, painful and otherwise) experiences of impairment. At the vanguard of national grassroots organisation of disabled people as well as formation of Disabled People’s International (DPI), he also chaired the first course in the world on ‘handicap’ and started the disability arts movement in the United Kingdom (Sutherland 2011). Finkelstein was a ‘white’ middle class South African and while he faced marginalisation from society, this was very different from the experiences of most ‘black’ South Africans who had to deal with enforced poverty, lack of rights, indiscriminate violence and institutional racism everyday (Howell, Chalklen & Alberts 2006; Watermeyer 2006). This mobilised him and other activists to organise more holistically around disability to create advocacy networks across the world.

### Disability rights activism

The anti-apartheid, black consciousness and student movements in South Africa all played big roles in inspiring disability activism and self-organisation of the late 1970s and throughout the 1980s (Howell et al. 2006). The 1980s proved pivotal for disability activism in terms of international attention and South African support. Howell et al. (2006) argue that visits of South African disability leaders, like Mike Du Toit, to the 1980 Rehabilitation International Conference where activists rejected medical professional control and interpretations of disability, was foundational. Until that point, disability was viewed as a medical problem needing rehabilitation. The conference questioned that understanding of disability and the emphasis was placed on common forms of oppression instead. Mike Du Toit was clear that this does not mean a rejection of professionals nor rehabilitation but a realisation of bigger needs and entitlements of citizenship, as well as removal of all barriers (Coleridge 1993; Howell et al. 2006). Disabled people walked out of the conference and set up their own organisation, which become DPI. This was important in uniting disabled people across differences of impairment, class, ethnicity, gender and so on, and in advocating for international collaboration and change against discrimination to reclaim their rights and places in society. The year 1981 was the UN year of disabled people, 1982 heralded Disabled People South Africa as begun by disabled activists and 1983 heralded the UN decade of disabled persons. Thus, the 1980s began cross-sectional national disability activism but also connected it to international institutions, legislation and support. In spite of the national activism of the 1970s and international activism of the 1980s, disability was still viewed as a specialised issue and not really connected to the broader landscape of inequalities in South Africa. In many ways, an understanding of *ubuntu,* as moving away from artificial differences between people and as enabling an environment of dignity was not brought to the fore.

### A shared common humanity?

As stated above, disability rights activists were involved in the anti-apartheid movement, which influenced the social model as well as the ways in which disability as experience of double or even triple oppression occurs. In order to include and link disability to other forms of human rights in the post-apartheid policy landscape of the 1990s, activists also began advocating for disability rights. The South African Disability Rights Charter became pivotal to the movement, but when there was political inaction, disabled people took over central Durban in 1992 to protest and get their rights on the political agenda (Watermeyer 2006). The fact that they had to do this speaks to the way in which the damage done to *ubuntu,* through a history of colonisation and apartheid that emphasised differences between people instead of uniting them, was never fully interrogated. As such, the legacy of Nelson Mandela in actually putting into practice *ubuntu,* in its holistic form of democratic interdependence, was never engaged with politically. If I am, because we are, then ensuring your well-being, dignity and rights as citizen, will also ensure mine.

This meant that splintered rights activism in South Africa often faced an uphill battle to connect citizenship to broader debates around how to put *ubuntu* into wider social, political, economic and environmental actions, rather than just historical reconciliation. In this way, social injustices and inequalities, while visible to people, were treated as invisible in broader debates. If we examine the fight for greater democracy and equality in South African society and take human immunodeficiency virus infection and acquired immune deficiency syndrome (HIV/AIDS) activism as an example, disability was initially excluded from such debates. The new ‘moral politics’ of HIV/AIDS (Robins 2006) emphasised collective responsibilities for individual human life against much political and economic pressure, but this did not initially include disabling symptoms of HIV/AIDS nor the disabled community (Hanass-Hancock & Nixon 2009). It was only from 2000 onwards, when the political fight for generic anti-retrovirals for all South Africans became successful that people began to think of cross-sectional issues between HIV/AIDS and disability in terms of chronic or ‘episodic’ disablement, medical and rehabilitative needs across the life course (Hanass-Hancock & Nixon 2009). By 2007, the disability resources that had been built up professionally, such as community-based rehabilitation, became relevant to HIV/AIDS testing and treatment, and the needs of disabled people linked to HIV/AIDS were taken into account in the Africa Disability and HIV & AIDS Campaign (Hanass-Hancock & Nixon 2009). While these changes occurred during the African Decade of Disabled People (1999–2009), disability was still viewed as a demarcated issue and not a part of enabling a human diversity as argued in African understandings of *ubuntu*. Human diversity entails interrogating the political and economic environment and demanding social justice. In order to understand this common humanity and shared diversity, it is useful to examine African environmental activism and what the concept of responsibility for others and shared caring encompasses.

## ‘Silence would be treason’: Environmental justice in Nigeria and Kenya

When thinking about protracted conflicts, terror and violence in Africa, connections are often made to its rich natural resources and polemical debates often centre around the ‘resource curse’ and tensions between states, (inter)national organisations and transnational corporations (Humphreys, Sachs & Stiglitz 2007). If we think about conceptions of *ubuntu* as inclusive of *ukama* and disability as a part of ecological diversity (Le Grange 2012; Murove 2004), Africans have often been ahead of their time in protesting and inciting activism to protect the environment and differing ecological homelands of their people - often from corrupt regimes and large multi-national corporations. If we take the environmentalists Ken Saro-Wiwa and Wangari Maathai as examples, we note that at the heart of their actions is *ubuntu.* Both understood the ethics of interconnectedness between the individual and natural environment to ensure people’s well-being, dignity and livelihoods.

Ken Saro-Wiwa, the Nigerian writer, businessman and environmental activist, was one of the first to examine the external and internal political conflicts that lay at the heart of these complex relationships, problematising Nigerian state kickbacks and thus legitimacy. Twenty years ago, he launched one of the most successful ecological non-violent protest movements, on behalf of the indigenous Ogoni people and their homeland, against the national and international oil companies like Shell (Brittain 2015; Pegg 2015). Ettang (2014) argues that at the heart of non-violent movements in Africa is the concept of ‘community’ found at the heart of *ubuntu* but I argue that it is the social ethics for the wider environment and *ukama* that underlies a common humanity. If those social ethical bonds that allow a common humanity are violated and oppression occurs, people like Saro-Wiwa and Wangari Matthai because of their interconnectedness take on the collective responsibilities of practices and discourses of justice to restore this and engage in not only healing but also rebuilding the environment.

While Saro-Wiwa fought against the environmental destruction that the Shell oil company, the British and the Nigerian government were complicit in, it was because of the negative effects it had for human life. He was galvanised by the physical and spiritual environmental threat Shell and the Nigerian government posed to the Ogoni people and their homeland. He and other Ogoni leaders were founders of the Movement for the Survival of the Ogoni People (Mosop) and organised successful mass indigenous demonstrations against corporations like Shell. This brought the environmental destruction happening in Nigeria to the world stage, but also advocated for the rights of indigenous people with the declaration of the Ogoni Bill of Rights. Saro-Wira’s last letters from prison before he was hanged by the Nigerian military government, alongside eight other Ogoni leaders, in 1995 are found in a book entitled ‘Silence would be treason’ (Corley, Fallon & Cox 2013). In those writings, he argues that he stands on the right side of a collective human history and that by keeping silent he would be complicit with the destruction of Ogoniland, which would be treason. The United Nations Environmental Programme (UNEP) published a report in 2011 that illustrated how destructive oil pollution had been and that clearing this up would take over 30 years (UNEP 2011). Fundamentally, UNEP (2011) agreed with Mosop and a one billion dollar clean-up operation of the Ogoni homeland finally began in 2016 with the support of the Nigerian government. Saro-Wira took on the responsibilities of collective discourses and actions to ensure *ubuntu* when it was ignored nationally and internationally.

Likewise, the Kenyan environmentalist, academic and politician, Wangari Maathai, early on in her book about the Green Belt Movement (GBM), describes how the UNEP being founded in Nairobi in 1972 was of pivotal importance because national governments were more focused on economic gains and tended to exclude environmentalism politically from those activities (Maathai 2004a). While the UNEP, and the nongovernmental organisations (NGOs) they involved, were mainly from the Global North, they began to involve southern activists and African women who had been excluded from such debates (Maathai 2004a). The GBM worked with Kenya’s National Council of Women mainly to empower rural women by planting trees for reforestation starting in 1977. Maathai states that she noticed that many rural women were identifying problems of environmental degradation but did not understand the causes and how that linked in to experiences of lack of food or unemployment (Maathai 2004a). For Maathai, while land and access to resources was tied up in/with decolonisation, land-grabbing, deforestation and environmental destruction of commercial farming were also connected to an unscrupulous government. She (Maathai 2010:16) argued that the values that sustained the GBM ‘defined a common humanity’ in upholding the life and well-being of women and their rural communities. Furthermore, she stated that in ‘degrading the environment, we degrade our selves’ (Maathai 2010:17). For her, there was an interconnectedness between educating women about the environment, sustainability and inter-generational livelihoods that need to be protected for real democracy to occur. Hence, Maathai’s idea of democracy encompasses the responsibilities of citizens for the protection of ecological diversity and an understanding of the importance of this for our humanness and future freedoms.

The work of the GBM came into conflict with the Kenyan regime in the 1980s and 90s when Maathai’s influence, campaigns against projects on protected land and pro-democracy protests began to rise (Maathai 2004a). While targeted by the government and jailed briefly along with pro-democracy activists, she began to engage in hunger strikes that gathered international attention and were supported by mothers of imprisoned activists (Maathai 2008). During the move to greater democracy in Kenya in the 1990s, she also tried to prevent ethnic violence by planting peace trees but felt that she had to engage in the political system to try and change things from within (Maathai 2008). This was to ensure continued education about environmental sustainability and the impact of postcolonial appropriation of resources by those in power in complicity with multi-nationals.

Maathai was the first African woman to be awarded the Nobel Peace Prize in 2004 and in that speech she again makes links between sustaining the environment, democracy and peace to ensure human dignity (Maathai 2004b). The most important part of the speech repeats the idea that it is Africans who will find solutions to the problems that they experience and that they have a crucial ‘cultural biodiversity’ to protect and enable them (Maathai 2004b). However, in order to do so, she argues they have to stand up to bad governance and that the ‘responsibilities of civil society’, to fight for injustices occurring against the environment, cannot be evaded because of their importance to create ‘cultures of peace’ (Maathai 2004b). Both Saro-Wiwa and Maathai encompass what *ubuntu* in discourse and practice entail as a humanist philosophy and ethical social practice connecting people, animals and the earth. As such, they broaden concepts of humanity to include ecological interdependence (*ukama*) and explain why protecting that diversity becomes necessary for African livelihoods and futures. In their understandings of humanness, they include our place within a physical and spiritual ecology that has to be culturally enabled.

## Discussion

The above examples, from activists based in south, west and east Africa, are illustrative of how far we have to go to truly implement *ubuntu,* not only in theory but also in truly restorative social ethical practices that encompass ecological diversity as part of humanness. Africans have begun to take the first steps to understand what *ubuntu* could mean, decolonise from a past and present history of violence and translate it in practice. An African model of disability that encapsulates *ubuntu* is correlated to how Africans have illustrated this social ethics of a common humanity in their grassroots struggles against oppression and disablement in the 20th century (Ettang 2014). This illustrates how disability activism and research in Africa can be understood as wider in focus than encountered in the Global North (Chataika et al. 2015; Mji et al. 2011; Opini 2016). In terms of learning from practices and discourses of decolonisation and setting differing disability agendas, Africans have their own histories and examples, to take as epistemological and ontological models to inform their understandings of disability, even in transnational and hybrid forms (Comaroff & Comaroff 2012; de Santos 2015). It also illustrates how *ubuntu,* as social ethics, has mobilised individuals and communities against injustices and the illusionary cohesion of the group (Louw 2006), to restore humanity and uphold individual life and well-being.

In comprehending what *ubuntu* means, the raising of a consciousness of oppression, disablement and legacy of colonial and present violence are interwoven. In African disability studies, more attention is needed to understand how colonial and postcolonial violence become linked to present-day oppression, disablement and loss of life through processes of direct, cultural and structural violence (Galtung & Fischer 2013). Using *ubuntu* we can ask why the social responsibilities of ethical actions are enabled or disabled individually, socially, by the state or structurally. For instance, while a mother from Ghana might attribute socio-cultural reasons to why her child with mental health issues is a ‘witch’ and needs prayer as a cure, the community must begin a dialogue to understand why *ubuntu* is not socially functioning for that mother and child? Socio-cultural and spiritual responses are also dynamic and we can begin to examine in whose interests culture has been negatively appropriated and what functions it serves for people? In Ghana, there is structural colonial and postcolonial violence in the lack of government education, access to medical treatment, care and choices linked to people’s mental health needs. That lacuna, in a context of poverty and stigma, is filled by Pentecostal churches and prayer camps who make money through warehousing children and adults with mental health issues, often leading to direct human rights abuses, suffering and violence (HRW 2012). *Ubuntu* does not place individual blame on a child, nor mother, but asks why a community, institution or state is failing in its compassionate responsibilities towards upholding respect for human diversity, who is filling the gap and why and what can be done to change such discourses and practices.

Decolonising disability in terms of *ubuntu* is not without risks because of the rewards and profit attached to oppression. Colonial and postcolonial violence illustrated the political difficulties and real dangers to embodiment of raising consciousness of any form of oppression but also how violence can be read as an illustration of disablement or lack of well-being and violation of humanity in a society. In many countries in Africa, differing national and international elites now use practices of violence and discourses that disavow diversity (i.e. by using ethnicity, tribalism, religion, gender and even *ubuntu*) to ensure control over resources and people to politically and economically enrich themselves. How do such longstanding inequalities and multiple forms of discrimination become linked to experience of disability? How do they affect the current issues that Africans are grappling with such as education, employment (formal and informal), corruption, violence, poverty and rise of extremism (such as Boko Haram in Nigeria) and securitisation. How does this affect disability activism, grassroots organisation, civil society and links to international institutions and advocacy when the state, international organisations and multi-nationals are predatory or function along their own interests? Does this mean that disability needs to be about ensuring greater *ubuntu* in society in a wider sense of diversity than impairment? What about in places where urbanisation has led to the creation of mega-cities and a rising middle class? What does *ubuntu* and disability entail in terms of ensuring an urban and spiritual sustainability?

If what disablement and oppression mean in the African context ties into the ascription of a difference that is threatening a common humanity, this entails that disability is also a more holistic concept then how it is understood in the Global North. If I *am* through the *otherness* and diversity of another (Eze 2008; Louw 1998), this does not deny feelings of ambivalence, pain or disgust (Livingston 2008) but locates them as part of the complexity and nuance of disability. It also calls into question the ascription of impairment as disability instead of diversity? What is at issue is what individual moral actions or restorative politics we engage in against disablement and oppression. Examining disability history in the African context illustrates that disabled people have always been part of a visible fight for justice and rights but that disability is still viewed as specialised individual medical issue because of colonial and postcolonial influence. The intersections between HIV/AIDS and disability revealed how important disability resources and knowledge can be but disability is not understood enough on an activist, practice or policy level. Linked is the false idea that disability rights have nothing to do with greater justice and peace in society. This totally counters the philosophy of *ubuntu* and functions to make invisible the contributions of disability activism and studies. Furthermore, *ubuntu* reveals that disablement can be physical, social and spiritual and lies in our understandings of what threatens the social order that allows the interpersonal relationships that let us be fully human. When making visible the invisible injustices that disable us, we have not really paid enough attention to how intersectionality as a concept also encompasses more diversity than just impairment orientated towards gender, age, ethnicity and so on. If moral disablement because of negatively perceived difference is a salient feature of disability, it also becomes important to understand how that changes and if and how it becomes connected to injustices that people experience. It also places certain people at social risk or wealth because of what it reveals about diversity of how embodiment is understood spiritually both in negative and positive ways. This opens up a concept of disability linked to a biodiversity and cultural diversity that is not just predicated on bodily types of visible or invisible impairment found in the Global North. Similarly, if that diversity is experienced as inhuman and a society cannot engage in the moral actions to ensure a change to accommodate *ubuntu,* then, learning from history, we will begin to see a fight for not just a restorative but a transformational disability politics.

*Ubuntu* locates disability politically within the wider environment and practices of sustainability, which are now important to the post-2105 agenda, CRPD and the (UN) Sustainable Development Goals linked to climate change. The links between disability, rights and ecology in the African context have not been given much attention despite the fact that there are links between environmental degradation and disablement. The Nigerian and Kenyan examples illustrate how Africans have been at the forefront of environmental activism, indigenous rights and ensuring claims to homelands. While they welcomed and used international support, they found ways in which to advocate using their own ideas and they did not need decolonisation. Why is this not happening linked to international organisations, funding and legislation linked to disability and the CRPD? While the CRPD continues to function like a straw man that is never seriously enforced, a different kind of political action linked to social justice seems to be evolving in line with *ubuntu*. It is noteworthy that environmental actions advocate repair of the natural environment and this includes the well-being of the ecosystem (plants and animals) in what encapsulates humanity. What this biodiversity could mean for disability and *ubuntu* remains relatively unexplored practically, for example, in terms of our relationships to animals, plants and technology. Within disability discourses, notions of repair and reparations for creation of physical and/or mental ill health, chronic conditions, impairment and future generational disablement linked to environmental social justice will gain greater credence, especially with the creation of, for example, biobanks for genomic research in Africa (see Staunton & Moodley 2013). Will such efforts lie in tension with the protection of the cultural biological diversity of impairment or will *ubuntu*, as social ethics, be interpreted in a differing way with regards to disability?

While the CRPD has a rich mandate and could be translated in terms of *ubuntu*, disability studies have mainly functioned on a state level or examined lack of inclusion and barriers in institutions or services that leave families and communities, and primarily women and children, to take on caregiving tasks. This has neglected the impact of globalisation and how transnational companies, institutions and services perpetuate inhuman relations or what broader forms of caregiving in terms of animal or ecological relationships could mean and how to ensure their enablement. Similarly, disability studies have examined direct impairments caused by arms- or conflict-related violence but not indirectly linked to degradation of ecosystems or impact of climate change. For example, disability could also be linked to desecration of ancestral lands, loss of forests or extinction of totemic animals – illustrative of how embodiment is linked to ecological diversity as cultural heritage and humanness. Instead, disability is becoming both a restorative and transformational politics by ignoring the ineffective state, for example, in South Africa, where mainly former mine workers have been suing transnational mine companies for breaches of health and safety resulting in loss of life, chronic ill health and disability. In the African context, such actions against injustice and links to disability as impairment, will only grow and move towards, for example, factory conditions, child workers in cocoa plantations, or the prison–industrial complex. Examining the links between environment and disability will also become inclusive of ‘suffering’ as affecting diversity; in slums, because of a lack of formal and informal opportunities of employment and then move on to questioning lack of infrastructure to ensure cultural and ecological equality, thus attacking the political and economic promises on an international policy level. Within an African focus on disability as *ubuntu*, the dignity of the otherness of another and respect for that diversity is at its heart. Anything that threatens a common humanity and individual humanity will lead to a quest for dignity through discourses and practices against this injustice. How Africans will interpret this in their terms, for present and future challenges, remains to be seen. It is a challenge that disability studies is taking up in keeping with the dynamism of *ubuntu* and how we understand what it means to be human.
